# Case of Ununited Fracture of the Forearm of Four Years Standing, Successfully Treated

**Published:** 1842-09-24

**Authors:** 


*Case of Ununited Fracture of the Forearm of four years standing, successfully treated.* By Charles S. Tripler, M. D., Surgeon U. S. Army.

In the September No. of the "Maryland Medical and Surgical Journal," this interesting case is reported at length. There had been fracture of both bones of the left forearm, the radius being "broken within two inches of its humeral extremity, and the ulna at one-third of its length from the elbow." The limb was useless, except in raising weights in the direction of its axis. The false joint of the ulna possessing most motion, it was supposed that if osseous union were effected in that bone, the arm might be useful for ordinary purposes. Forcible opposition was employed, and although the patient suffered a great deal, and frequently removed the apparatus, it was successful in eleven weeks. The mobility in the false joint of the radius was still so great as to demand an operation, and excision was resorted to, and was successful, in spite of several untoward accidents, in *six* weeks.

